# C-terminal binding proteins 1 and 2 in traumatic brain injury-induced inflammation and their inhibition as an approach for anti-inflammatory treatment

**DOI:** 10.7150/ijbs.42109

**Published:** 2020-02-04

**Authors:** Hong Li, Caiguo Zhang, Chunxia Yang, Melanie Blevins, David Norris, Rui Zhao, Mingxia Huang

**Affiliations:** 1 Department of Dermatology, University of Colorado Anschutz Medical Campus, Aurora CO 80045, USA; 2 Department of Psychiatry, First Hospital of Shanxi Medical University, Taiyuan, China; 3 Department of Biochemistry and Molecular Genetics, University of Colorado Anschutz Medical Campus, Aurora CO 80045, USA

**Keywords:** CtBP, proinflammatory transcription, neuroinflammation, microglia activation, traumatic brain injury

## Abstract

Traumatic brain injury (TBI) induces an acute inflammatory response in the central nervous system that involves both resident and peripheral immune cells. The ensuing chronic neuroinflammation causes cell death and tissue damage and may contribute to neurodegeneration. The molecular mechanisms involved in the maintenance of this chronic inflammation state remain underexplored. C-terminal binding protein (CtBP) 1 and 2 are transcriptional coregulators that repress diverse cellular processes. Unexpectedly, we find that the CtBPs can transactivate a common set of proinflammatory genes both in lipopolysaccharide-activated microglia, astrocytes and macrophages, and in a mouse model of the mild form of TBI. We also find that the expression of these genes is markedly enhanced by a single mild injury in both brain and peripheral blood leukocytes in a severity- and time-dependent manner. Moreover, we were able to demonstrate that specific inhibitors of the CtBPs effectively suppress the expression of the CtBP target genes and thus improve neurological outcome in mice receiving single and repeated mild TBIs. This discovery suggests new avenues for therapeutic modulation of the inflammatory response to brain injury.

## Introduction

Traumatic brain injury (TBI) is a leading cause of death and disability among children and young adults in the United States 1-3. It can be classified as mild, moderate and severe based on neurological injury severity. Severe TBI is a well-established risk factor for several diseases of the aging brain, including amyotrophic lateral sclerosis, Alzheimer's disease and Parkinson's disease 4. The mild form of traumatic brain injury (mTBI, also known as concussion) accounts for >80% of all TBIs 5, 6. The majority of mTBI patients experience full recovery within a couple of weeks to months. A small minority of these patients (10-20%) experience persistent postinjury symptoms 7-9. Disturbingly, even a single mTBI induces pathophysiological changes in the brain that can be detected in both acute and chronic phases postinjury 10-12. Athletes and military service members are especially vulnerable to multiple mTBIs, raising concerns about cumulative or long-term effects of recurrent insults 8, 13. In effect, repeated mTBI has been linked to late-onset neurodegenerative disease development, such as chronic traumatic encephalopathy 14, 15. There are currently no FDA-approved drugs specifically for TBI.

TBI elicits a distinct inflammatory response in the central nervous system (CNS) that involves both local and peripheral immunocytes 16-18. The acute phase of the injury begins within minutes of the primary mechanical damage to the brain tissue. Microglial and astroglial activation can persist for months and years after TBI and can affect CNS neurodegeneration. In mTBI, diffuse axonal injury, often resulting from impact/acceleration forces, leads to the release of danger signals such as damage-associated molecular patterns and alarmins from the stressed axons 19, 20. These signals induce the rapid activation of resident microglia, which upregulate the expression of an array of inflammatory mediators to amplify the immune response and recruit peripheral immune cells to the damage site. Excessive inflammation causes cell death and tissue damage and in the chronic phase may contribute to neurodegeneration 21. There is a compelling need for effective neuroprotective interventions that improve functional outcomes in TBI patients 22, 23. In spite of its recognized importance in the regulation of inflammatory responses, inhibition of NF-κB showed a limited neuroprotective effect in experimental models of TBI 24, 25. As such, the discovery and development of new modulators of brain injury-mediated neuroinflammatory response are the subject of intense pharmaceutical efforts.

C-terminal binding protein (CtBP) 1 and 2 are paralogous transcriptional coregulators that repress diverse biological processes by directly binding transcription factors (TF) and recruiting chromatin-modifying enzymes to target gene promoters 26, 27. These two proteins exhibit a remarkable structural similarity to D2-hydroxyacid dehydrogenases, including an N-terminal substrate binding domain, a central NAD(H)-binding domain and a C-terminal extension 28. They also contain a hydrophobic cleft within the N-terminal substrate binding domain known as the PXDLS-binding site and a surface groove within the central NAD(H)-binding domain that recognizes the sequence RRTGXPPXL (RRT motif) 29-31. These two unique structural elements constitute the majority of CtBP interactions with transcription factors and chromatin modifying enzymes such as histone deacetylases, acetyltransferase, methyltransferases and demethylases 32-35. NAD(H) facilitates the dimerization of the CtBPs and promotes their transcriptional corepression function 36, 37. The CtBPs, which were originally identified through their binding to the adenovirus E1A oncoprotein 38, have been demonstrated to regulate multiple transcription factor networks with roles in tumorigenesis and cell survival 26, 39, 40. Inhibition of the interaction between the CtBPs and their PXDLS-containing target proteins by the peptide Pep1-E1A has been shown to block transcriptional repressor activity and mitigate oncogenic phenotypes in mice 41. On the other hand, the small molecule MTOB that inhibits the dehydrogenase activity of the CtBPs attenuates the growth and self-renewal of cancer stem cells *in vitro* and decreases tumor burden in a mouse model of colon cancer 42-44. In addition, it is postulated that the CtBPs also function as context-dependent transcriptional coactivators 34, 45, 46. In this report, our results indicate that CtBP1 and CtBP2 increase the *in vivo* transcription of prionflammatory genes in response to mTBI; reveal that the mechanism underlying both local and systematic induction of these CtBP target genes operates in an impact energy dose- and time-dependent manner; and indicate that Pep1-E1A and MTOB inhibit the transactivating activity of the CtBPs, reduce microglia activation and ameliorate neurological deficits following single and repeated injuries in mice, thus demonstrating the potential utility of these agents to modulate the immune response to head injury.

## Materials and Methods

### Animals

All animal experiments were reviewed and approved by the University of Colorado Anschutz Medical Campus Institutional Animal Care and Use Committee (protocol B-114116(12)1E) and performed in accordance with relevant guidelines and regulations. C57BL/6 mice were group housed in environmentally controlled conditions with 12:12 h light:dark cycle and provided food and water *ad libitum*.

### CHIMERA TBI

Mice at 16-20 weeks old of age (body weight 20-30 g) were randomly assigned to sham, injury, or injury with treatment groups. Animals were anaesthetized with isoflurane (induction: 4.5%, maintenance: 2.5-3%) in oxygen (0.9 L/min) and underwent TBI or sham procedures as described 47. The average total duration of isoflurane exposure for the whole procedure was 3 to 4 min. For single TBI experiments, mice received a closed-head impact from a pneumatically driven 50 g steel piston that was calibrated to deliver 0.5 to 0.8 J of kinetic energy at the point of impact. An impact of 0.5 J energy was defined as the low threshold for producing CHIMERA-delivered mild brain injury in a previous dose-response study 48. In the repeated mTBI experiment, mice received two successive 0.5 J impacts spaced 24 h apart. The sham group underwent all procedures except for the impact. For receiving head injury, the animal was placed in a supine position in the holding bed with the top of its head placed flat over a hole in the head plate. The top of the animal's head was aligned using crosshairs so that the piston strikes a circular area with a radius of 2.5 mm surrounding the bregma on the vertex of the head. In the control experiment to distinguish between the local and systemic effect of CHIMERA-delivered impact (see Figure S2), animals were held in place such that the animal's back or one ear was positioned over the hole of the head plate to receive the piston-delivered impact.

### Administration of CtBP inhibitors

Preparation of the PLDLS-containing Pep1-E1A and the PLDLS-to-PLDEL mutant (Pep1-E1A^Mut^) peptides were as previously described 41. Peptides (6 mg/mL) and MTOB sodium salt (Sigma, K6000; 425 mg/mL) were dissolved in water. NSC95397 (Sigma, N1786; 5 mg/mL) was dissolved in DMSO. The stock solutions were diluted in PBS solution (vehicle) to desired final concentrations freshly before use. Pep1-E1A was delivered at 2 mg/kg dosage, 4-methylthio-2-oxobutyric acid (MTOB) sodium salt at 860 mg/kg and NSC95397 at 0.75 or 1.5 mg/kg with intraperitoneal (i.p.) injection.

### Behavioral analyses

The latency to righting reflex (a.k.a. loss of righting reflex, LRR), a surrogate measurement for loss of consciousness after brain injury, is used as a behavioral indicator of injury severity 49. The LRR duration was recorded as the interval from the time isoflurane exposure was discontinued till the time at which the animal spontaneously righted itself from a supine position 47. Post-traumatic neurological impairments were evaluated using a 10-point neurological severity score (NSS) paradigm, which evaluate mice in performance of ten tasks: circle exiting, seeking behavior, gait pattern (monoparesis or hemiparesis) and paw grip, straight walk, startle reflex, beam (7 mm × 7 mm) balancing, beam walking on a flat surface of 3, 2 and 1 cm width, and round stick (5 mm in diameter) balancing 50. One point is given for the lack of a tested reflex or the inability to perform the task, with a maximal NSS of 10 points indicating the failure of all tasks.

### Tissue collection, histology, and immunofluorescence

Mice were anesthetized with isoflurane via a nose cone. Blood samples were collected by cardiac puncture into EDTA-coated tube to prevent clotting. Leukocytes were collected by two rounds of 10-min incubation in a red blood cells lysis buffer (0.8% NH_4_Cl, 0.084% NaHCO_3_ and 0.037% EDTA) followed by centrifugation at 2500 × g for 10 min, all at room temperature. The pelleted white blood cells were frozen in liquid nitrogen and stored at -80°C until RNA extraction. The animals were transcardially perfused with ice-cold 0.8% NaCl solution for 4 to 5 min until the liver is cleared of blood. Mouse brain was obtained by decapitation of the perfused animal. For biochemical analyses, the brain was longitudinally hemisected, rapidly frozen in liquid nitrogen and stored at -80°C until RNA and protein extraction. For immunohistological analyses, mice were perfused with PBS followed by 4% ice-cold paraformaldehyde-PBS solution for 5 min. Brain tissue were post-fixed in 10% formalin-PBS for 48 h at 4°C, embedded in paraffin, and sectioned coronally to 40-μm thickness. The primary antibodies used for immunofluorescence imaging were: mouse anti-CtBP2 (1:200,BD, 612044), goat anti-CtBP2 (Santa Cruz, sc-5966, 1:100), rabbit anti-Iba1 (GeneTex, GTX100042, 1:200), rabbit anti-GFAP (Abcam, AB7260, 1:1000), mouse anti-NeuN (GeneTex, GTX30773, 1:100). Secondary antibodies used were IgG conjugated to Alexa Flour 594 or 488 (1:500, Invitrogen), biotin-labeled IgG (1:300, Vector lab), Streptavidin conjugated to Alexa Flour 594 or 488 (1:2000, Life Technology, S11227 and S11223). Mouse on Mouse (MOM) Detection kit (Vector Laboratories, BMK-2202) were used to reduce endogenous mouse Ig straining when using mouse primary antibodies on mouse tissues. All slides were counterstained with DAPI to visualize the nuclei.

### Cell transfection and LPS treatment

The mouse macrophage cell line RAW264.7 was obtained from American Type Culture Collection (ATCC, VA, USA). The mouse microglial BV2 cell line was a kind gift from Dr. Manisha Patel (University of Colorado Anschutz Medical Campus). Both cell lines were cultured in Dulbecco's modified Eagle's medium (DMEM; Corning 10017CV) supplemented with 10% fetal bovine serum (FBS, GEMINI, 100-500) and 1% penicillin/streptomycin (Corning, 30002CI) at 37°C with 5% CO_2_.

Cell transfection was carried out in 24-well plates as described 51. Briefly, RAW264.7 and BV2 cells under approximately 80% confluence were treated with 0.25% Trypsin-EDTA (Corning, 25-053-CI) and then transfected with Lipofectamine 2000 (Invitrogen, 11668019) in suspension with siRNAs specific to mouse *CtBP1* (UCUUCCACAGUGUGACUGCGUUAUUUU, 50 nM), *CtBP2* (GCCUUUGGAUUCAGCGUCAUAUUU, 50 nM), or both (25 nM each). The transfected cells were incubated in DMEM for 24 h, followed by treatment with 200 ng/mL LPS (Sigma-Aldrich, L3880) for 6 h, and harvested for RNA extraction.

### Primary microglia and astrocyte cultures and treatment with peptide and LPS

Mouse primary microglia and astrocytes were isolated from cerebral cortices of neonatal pups (P0 to P2-d-old) and cultured as described 52, 53, with minor modifications. The isolated cortices were washed with ice-cold PBS containing 0.1% BSA and 1× minimum essential medium (MEM) non-essential amino acids (Corning, 25025CL). Single cell suspension was made by repeated pipetting the trypsin-treated cortical tissue through a sterile 10 ml pipette ten times followed by passing through a 18G needle three times. The mixed cortical cells were passed through a nylon mesh cell strainer with 70-μm pores and plated on an uncoated T75 flask in DMEM (Corning, 10013CV) with 10% FBS (Gemini, 100-500). After the mixed glial culture reached confluency at 10 to 14 d post-plating, microglia were detached using an orbital shaker (220 rpm, 1 h), collected and re-plated in DMEM with 15% FBS. Oligodendrocyte precursor cells were removed by further shaking at 220 rpm for 6 h. The remaining astrocyte layer were detached by trypsin digestion and re-plated in DMEM with 10% FBS. The primary microglia and astrocyte cultures were >90% pure based on immunofluorescence imaging with antibodies specific to Iba1 and GFAP, respectively.

Cultured primary microglia or astrocytes were incubated with 20 μM Pep1-E1A or Pep1-E1A^Mut^ for 2h before the addition of 200 ng/mL LPS (Sigma-Aldrich, L3880). Cells were harvested 2 h after LPS treatment for RNA extraction. Anti-FLAG immunofluorescence staining was performed to monitor cellular internalization of the peptides as described 41.

### Reverse transcription and quantitative real-time PCR (RT-qPCR)

Total RNA was extracted from freshly harvested cells or frozen tissues using TRIzol^TM^ reagent (Invitrogen, 15596026) and analyzed by RT-qPCR. First-strand cDNA was reverse transcribed from 1.0 μg total RNA with oligo (dT) primers using Verso cDNA synthesis kit (Thermo Scientific, AB1453A). Quantitative PCR with SYBR green detection (Applied Biosystems, A25741) was performed using 1% of the reversely transcribed cDNA mixture on a BioRad CFX96 real-time detection system. Relative expression of individual genes were normalized to *ACTB* (β-Actin) expression using the 2^-ΔΔCt^ method 54. The sequences of all RT-qPCR primers were provided in Supplementary Table 1.

### Chromatin immunoprecipitation (ChIP)

ChIP experiments were carried out as described 55 with modifications. In brief, cells were cross-linked with 1% (w/v) formaldehyde for 15 min at room temperature before the cross-linking was quenched with 0.125 M glycine for 5 min. About 1 × 10^8^ cross-linked cells were lysed by sonication for 15 × 30 sec with 30-sec break on ice in 10 mL lysis buffer (0.1% SDS, 0.5% Triton X-100, 150 mM NaCl, 20 mM Tris-pH 8.1, 1 mM DTT, 2 mM EDTA, and with protease inhibitor cocktail freshly added before use; Roche, 11836153001). After centrifugation at 19,000 × g for 15 min, the supernatant was used for immunoprecipitation with 1/100 of the lysate (100 μL) set aside as the input sample. The remaining lysate was split into four equal parts and incubated with each antibody (mouse anti-CtBP1, BD Biosciences, 612042; mouse anti-CtBP2, BD Biosciences, 612044; mouse anti-p300 Santa Cruz, sc-48343; and mouse IgG_1_, Santa Cruz, sc-69786) overnight with rotation at 4°C. Protein A beads (RepliGen, 10-1003-03) were added to the lysate with rotation at 4°C for 4 h. The beads were washed 5 × 5 min with the lysis buffer. The precipitated DNA-protein complex was eluted into 300 μL 1 mM NaHCO_3_ and 1% SDS by incubation at 65°C for 15 min. After centrifugation at 19,000 × g for 1 min, the supernatant was transferred to fresh tubes, followed by supplementation with 5 M NaCl to a final concentration of 300 mM and incubation at 65°C overnight to reverse the cross-linking. Protein and SDS of both input and IP samples were removed through phenol-chloroform extraction and ethanol precipitation. The DNA of input and ChIP samples was resuspended in 100 μL and 400 μL H_2_O, respectively. For qPCR, 1.5 μL from each sample was used. For each antibody, relative ChIP signal was calculated as percent of input using the 2^-ΔΔCt^ method 54 and the ChIP signal of the LPS-stimulated cells were normalized to that of the non-stimulated control. The sequences of all ChIP-qPCR primers were provided in Supplemental Table S1.

### Western Blotting

Protein extracts were prepared from longitudinally halved mouse brain (~200mg) in 500μL homogenization buffer consisting of 10 mM Tris, pH 7.4, 100mM NaCl, 1mM EDTA, 1mM EGTA, 1% Triton X-100, 10% glycerol, 0.1% SDS, ​0.5% deoxycholate, and 1× complete protease inhibitor cocktail (Roche, 11836153001). Debris were removed by centrifugation at 18,000 × g for 5 min at 4°C. The supernatant fraction was quantified through use of the Bradford assay (Bio-Rad, 5000006). For each sample, 50 μg of total protein extracts was resolved by 12% SDS-PAGE and transferred onto a PVDF membrane for immunodetection. The membrane was incubated with antibodies specific to CtBP1 (BD Biosciences, 612042, 1:1,000), CtBP2 (BD Biosciences, 612044, 1:1,000), S100A9 (Santa Cruz Biotechnology, sc-58706, 1:1,000), NLRP3 (Santa Cruz Biotechnology, 134306, 1:1,000) and GAPDH (Sigma, G8795, 1:5,000) at 4°C overnight, followed by peroxidase-labeled appropriate secondary antibodies at room temperature for 1h. The membrane was developed using an enhanced chemiluminescence substrate (Millipore Corporation, WBKLS0500) and scanned with a ChemiDoc MP imager (Bio-Rad). Raw signal intensity for each band was measured using Image J software (4.0.1 version).

### Statistical Analysis

Plots were made and statistical analysis was performed using GraphPad Prism version 8.0 (GraphPad Software). Data are expressed as mean ± standard deviation (SD). In cell culture experiments the '*n*' values denote the number of experiments. Each independent experiment contained triplicate cultured wells.

## Results and Discussion

### CtBP1 and CtBP2 are required for the transactivation of proinflammatory genes in LPS-activated microglia and macrophages

**Figure 1:**
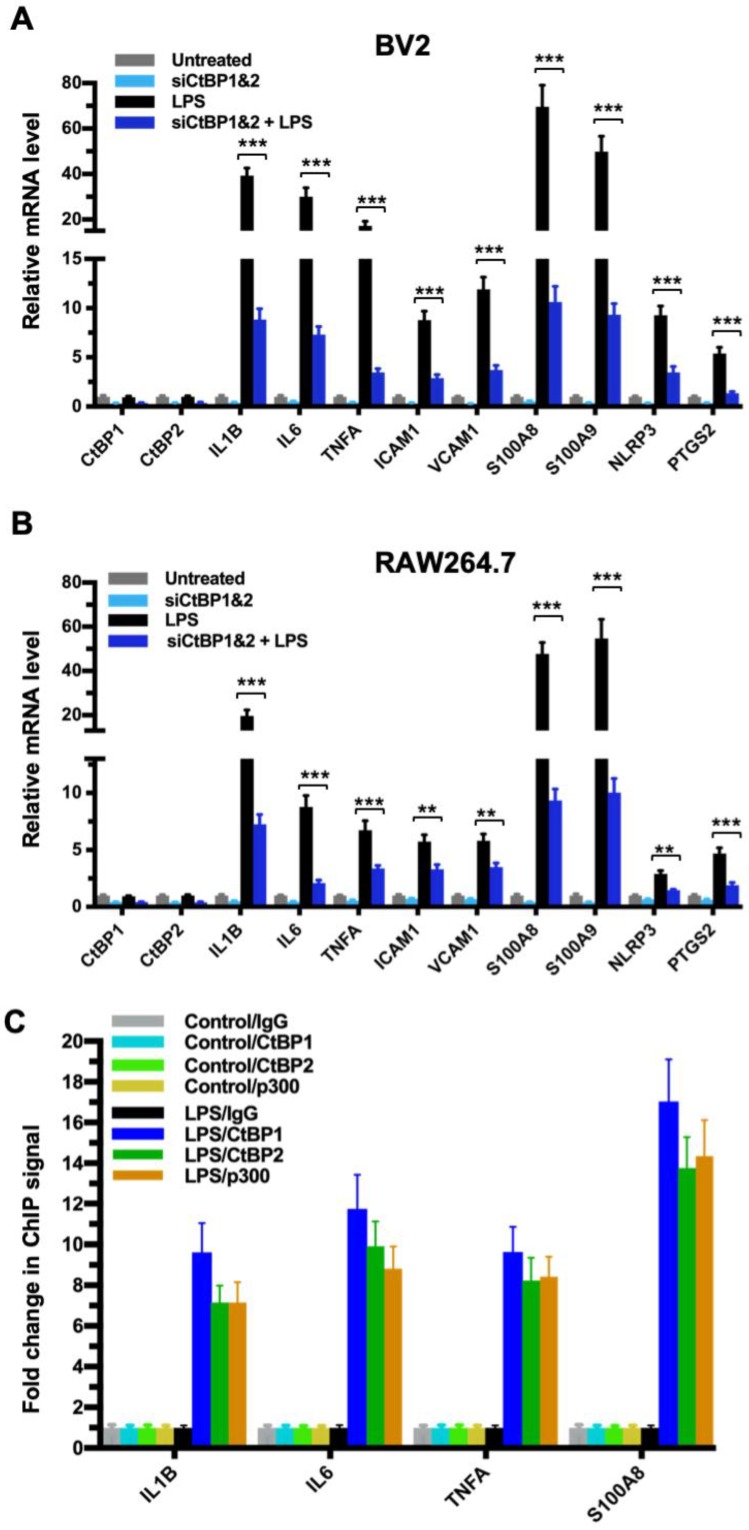
** CtBP1 and CtBP2 are required for LPS-induced proinflammatory gene expression in microglia and macrophage cell lines. (A and B)** Simultaneous knockdown of CtBP1 and CtBP2 suppresses mRNA expression of proinflammatory genes in LPS-activated microglia and macrophages. Murine RAW264.7 **(A)** and BV2 **(B)** cells were transfected with siRNAs specific for CtBP1 and CtBP2 or scrambled control siRNAs for a period of 24 h, followed by LPS stimulation (200 ng/ml) for 6 h. Total RNA was extracted and analyzed by the RT-qPCR. Relative mRNA expression was normalized to that of *ACTB* and depicted as fold changes vs. the scrambled siRNA transfected and non-stimulated control (Untreated). Data are presented as mean ± SD. *n* = 3; **p<0.01, ***p<0.001. **(C)** LPS induces increased binding of CtBP1, CtBP2 and p300 to the *IL1B, IL6, TNFA* and *S100A8* gene promoters. Chromatin fractions from control and LPS-treated BV2 cells were precipitated with antibodies specific to CtBP1, CtBP2 and p300. Bars represent fold changes of relative ChIP signals normalized to the respective controls.

To explore the role of the CtBPs in cellular regulation of the inflammatory response, we investigated their occupancy on the promoters of the 674 inflammatory response genes of the Gene Ontology GO:0006954 set 56, 57 through the analysis of CtBP ChIP-seq data obtained from non-stimulated breast cancer cells 58. We observed a moderate enrichment in the promoters of a select set of genes encoding proteins involved in proinflammatory response, including cytokines (e.g., IL-1β, IL-6, TNF-α), cell adhesion molecules (ICAM-1 and VCAM-1), alarmins (S100A8 and S100A9), inflammasome (NLRP3) and prostaglandin synthase (PTGS2). To assess whether the inflammation-induced expression of the above genes is CtBP-dependent, we evaluated the effects of siRNA-mediated simultaneous knockdown of *CtBP1* and *CtBP2* on the mRNA levels of these candidate genes in lipopolysaccharide (LPS)-stimulated microglia and macrophage using the reverse transcription and quantitative real-time PCR (RT-qPCR) method. As shown in Figures 1A and 1B, exposure to LPS induces the expression of all nine genes (3- to 70-fold increase) in the murine BV2 microglia and RAW264.7 macrophages. Silencing of the CtBP genes markedly decreases the LPS-induced gene expression, ranging from 40% to 85% (see Figures 1A and 1B), suggesting that CtBP1 and CtBP2 are involved in the upregulation of these genes in response to LPS activation. We also observed similar CtBP-dependent activation of the above nine genes in LPS-stimulated human THP-1 monocytes. Thus, CtBPs likely regulate the expression of the nine candidate genes in the human and mouse monocytes. To validate our analysis, we performed chromatin immunoprecipitation (ChIP) experiments in the LPS-activated BV2 cells to measure CtBP binding to the promoter regions of the four strongly induced genes (*IL1B, IL6, TNFA* and *S100A8*) as a testbed. Indeed, LPS stimulates a significant increase in the binding of CtBP1 (10- to 17-fold change) and CtBP2 (7- to 14-fold change) to the promoter regions of these genes (Figure 1C). We also found that the promoter recruitment of histone acetyltransferase (HAT) p300, a PXDLS-containing protein that binds CtBP 32, is intimately associated with CtBP occupancy at the four target genes (Figure 1C). Taken together, our findings indicate that CtBP1 and CtBP2 serve as transcriptional coactivators capable of enhancing LPS-induced expression of the nine aforementioned proinflammatory genes.

### Pep1-E1A suppresses the induction of CtBP target genes in LPS-activated mouse primary microglia and astrocytes

**Figure 2:**
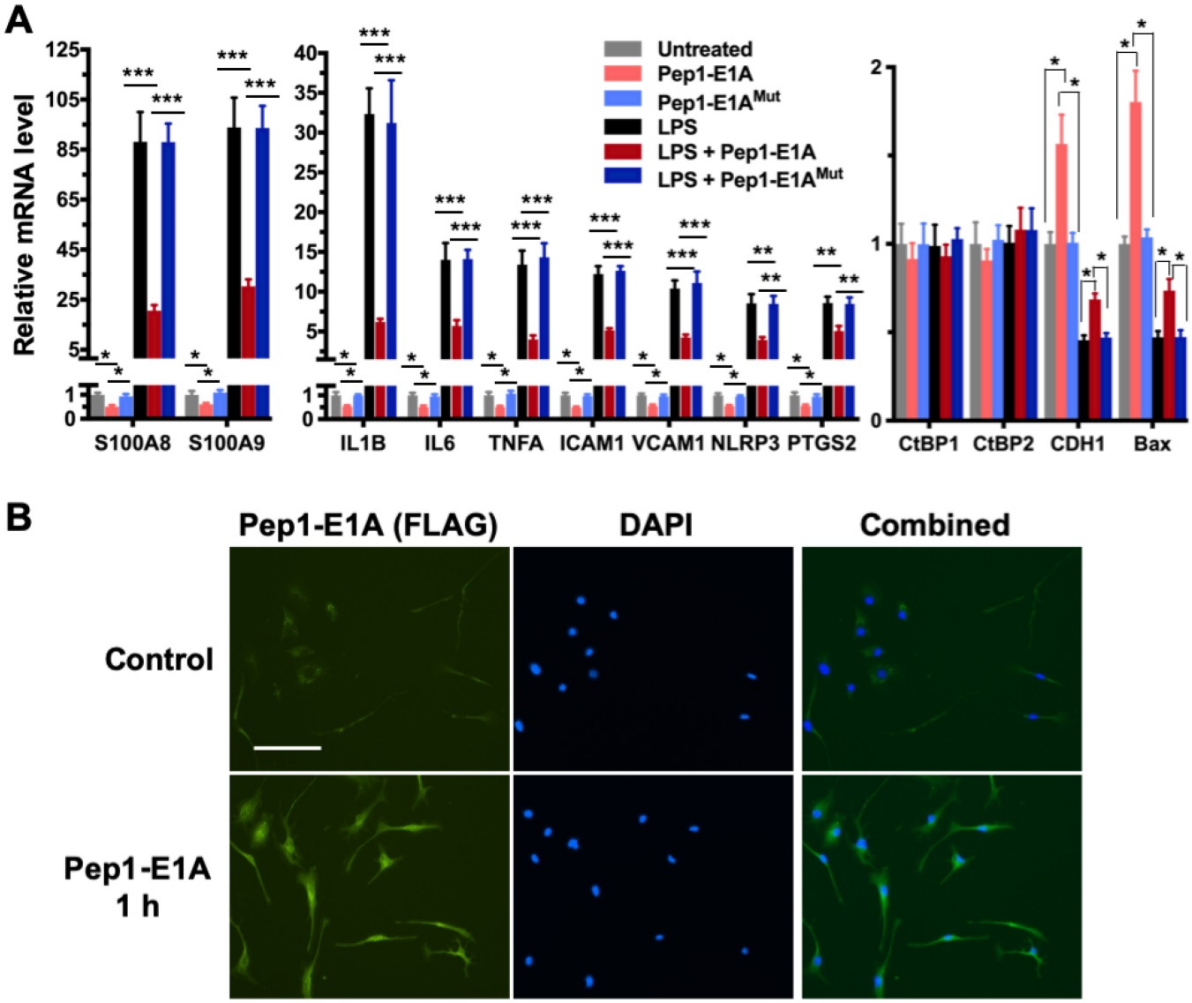
** Pep1-E1A suppresses the induction of CtBP-regulated proinflammatory genes in LPS-activated mouse primary microglia. (A)** Mouse primary microglia were incubated with 20 μM Pep1-E1A or the PLDLS-to-PLDEL mutant (Pep1-E1A^Mut^) peptide for 2 h, followed by LPS stimulation (200 ng/ml) for 2 h. Relative mRNA expression was depicted as fold changes vs. the untreated controls. *n* = 3; *p<0.05, **p<0.01, ***p<0.001. **(B)** Representative immunostaining images of Pep1-E1A internalization into cultured primary microglia after 1 h incubation with the peptide. Scale bar, 50 μm.

We have shown previously that the Pep1-E1A peptide disrupts the interaction of CtBP1 with the PLDLS-containing repressor ZEB1 in cancer cells and relieves the transcriptional repression of *CDH1* (E-cadherin) and *BAX* by the CtBPs (Blevins et al., 2018). To explore whether this peptide inhibitor can interfere with the transactivation function of CtBP1 and CtBP2, we preincubated mouse primary microglia and astrocytes with Pep1-E1A for 2 h prior to stimulation with LPS and measured mRNA levels of the above nine CtBP-regulated proinflammatory genes by RT-qPCR. As expected, the LPS treatment of both microglia and astrocytes increases the expression of these nine genes by 7- to 132-fold while reducing the expression of *CDH1* and *BAX* to approximately 50% of the levels seen in controls (Figures 2A and S1A). In contrast, Pep1-E1A pretreatment significantly suppresses the LPS-induced expression of the nine proinflammatory genes by 40 to 70%, and effectively relieves the repression of *CDH1* and *BAX* by ~50% (Figures 2A and S1A). We also note that Pep1-E1A pretreatment results in a 40% to 50% decreases in the basal mRNA expression of these proinflammatory genes in both primary microglia and astrocytes without stimulation with LPS, and causes a 57% and 80% increase in the basal mRNA levels of *CDH1* and *BAX*, respectively (Figures 2A and S1A). Consistent with previous study 41, Pep1-E1A internalized readily into microglia and astrocytes in culture (Figures 2B and S1B). We conclude that CtBP1 and CtBP2 play a crucial role in mediating the activation of the nine proinflammatory genes. These findings also indicate that the CtBPs exhibit a dual role in transcriptional activation and repression.

### Mild TBI causes dynamic changes in the induction of gene expression by CtBP1 and CtBP2 in both brain and peripheral blood leukocytes

**Figure 3:**
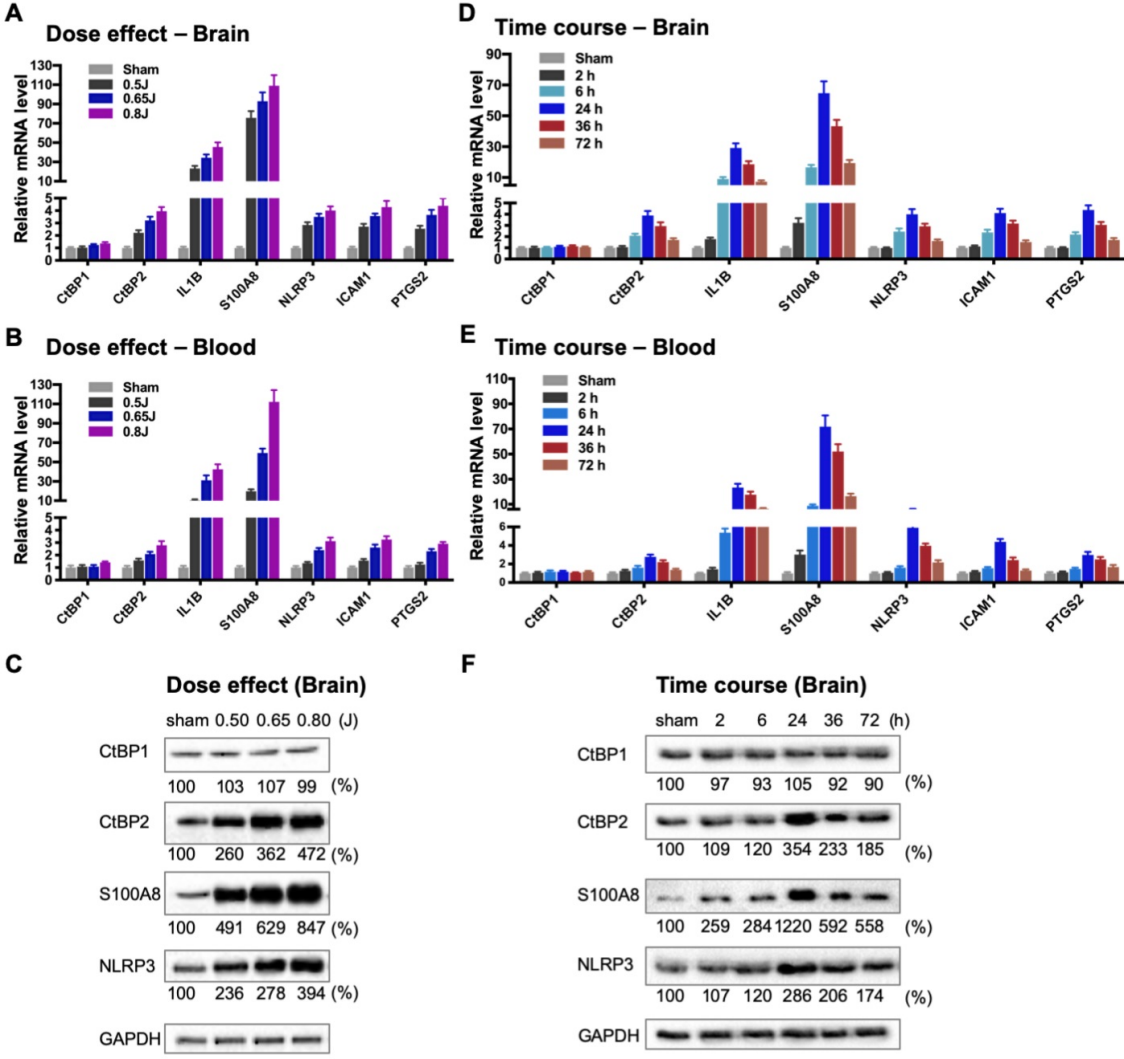
** Impact dose- and time-dependent induction of CtBP target genes in brain and peripheral blood leukocytes following single mild TBI. (A and B)** Mice (*n* = 5 per group) were subjected to a single head injury of varying impact energies and the brain **(A)** and peripheral blood leukocytes **(B)** were harvested for mRNA analysis 24 h after injury. Results were normalized to the sham group and are presented as mean ± SD. **(C)** Representative Western blots of the CtBP1, CtBP2, S100A9, NLRP3 proteins of the brain tissues from the dose-response groups. Relative protein expression was normalized to the loading control GAPDH and shown as percent change vs sham under the blots. **(D and E)** Mice (*n* = 5 per group) received a single head impact of 0.7 J energy and mRNA expression in brain **(D)** and blood **(E)** were analyzed at the indicated time points postinjury. **(F)** Western blots showing protein expression of the brain tissues from the time course experiment.

To directly evaluate the hypothesis that the CtBPs play a role in the regulation of the acute inflammatory response following mild TBI, we used a closed-head impact model of engineered rotational acceleration (CHIMERA) in mice to investigate the dose-response relationship between injury severity and changes in the expression of CtBP target genes *in vivo*. This mouse model was selected in the present study because diffuse axonal injury is the hallmark of inertial TBI 47. Animals received sham or a single head injury with the impact kinetic energy of 0.5 J, 0.65 J and 0.8 J, respectively. At 24 h postinjury, brain and blood leukocyte samples were collected to measure mRNA levels of the five CtBP-regulated proinflammatory genes identified above as well as those of *CtBP1* and *CtBP2* by RT-qPCR. The single brain injury leads to the increased expression of all five proinflammatory genes and *CtBP2*, in both CNS and systemic compartments, in an impact energy dose-dependent manner (Figures 3A and 3B). Specifically, the *NLRP3*, *ICAM1*, *PTGS2* and *CtBP2* genes are moderately upregulated in the mouse brain, with 2.2- to 2.9-fold change at 0.5 J, 3.2- to 3.7-fold change at 0.65 J and 4.0- to 4.4-fold change at 0.8 J. The *IL1B* gene is induced to a significant degree, with 23-, 34-, and 45-fold change at 0.5 J, 0.65 J and 0.8 J, respectively. The expression of *S100A8* is exceptionally high (76-, 93- and 109-fold change at 0.5 J, 0.65 J and 0.8 J, respectively). Remarkably, there is a strong, linear correlation between the mRNA expression levels of the above six genes in the mouse brain and peripheral blood leukocyte samples (Figures 3A and 3B). In parallel, we observed impact energy dose-dependent increases in the expression of the S100A8, NLRP3 and CtBP2 proteins in the injured mouse brain, as assessed by the Western blot method (Figure 3C). These data demonstrate the dose-dependent effects of mild brain injury on CtBP-mediated inflammatory response in mice. In addition, the upregulated *CtBP2* gene expression observed following mTBI has implications for the mechanism of CtBP-mediated transcriptional activation (see Figures 3 A-C).

To characterize the kinetics of the above head injury-induced gene expression, animals received a single impact of 0.7 J energy and were euthanized at different time points afterwards. The mRNA expression levels of the five proinflammatory genes and *CtBP2* started to increase in both brain and peripheral blood leukocytes as early as 2 h postinjury, peaked at 24 h, and then gradually decreased, but the level remained significantly higher than that in the sham control 72 h after injury (*p <* 0.05, Figures 3D and 3E). Western blot analysis also shows time-dependent changes in the expression of the S100A8, NLRP3 and CtBP2 proteins in the injured brain (Figure 3F). Moreover, the highest levels of these protein expression coincide with those of their mRNAs at 24 h postinjury (see Figures 3D and 3F). Our data suggest that the CtBPs may play a role in determining the timing and intensity of the inflammatory response to brain injury, consistent with cerebral and plasma proinflammatory cytokine profile following injury in human TBI patients 59, 60.

To address the question whether the TBI-induced proinflammatory gene expression observed in the peripheral blood leukocytes (see Figures 3B and 3E) is caused by the release of inflammatory mediators from the injured brain or the region of the skin and/or skull at the point of impact, three groups of mice received single head, back and ear impacts at 0.7 J energy, and their blood samples and skin tissues containing the site of the impact were collected 24 h postinjury. Moderate mRNA expression levels of the nine CtBP target genes identified in the present study were observed in the skin of the three injury groups, whereas the head injury group exhibits significantly increased mRNA levels of these genes in the circulating leukocytes relative to the other two groups (*p <* 0.001, Figures S2A and S2B). In particular, the most strongly upregulated genes *S100A8* and *S100A9* in circulating leukocytes display >95-fold change in the head injury group, and 6- to 8-fold change in the back and ear injury groups (Figure S2B). Our results suggest that the single mild head injury can evoke a systemic inflammatory response. Consistent with this argument, systemic inflammatory response syndrome is frequently observed in human patients with TBI 61. We therefore propose that CtBP1 and CtBP2 are potential mediators of both local and systemic inflammation in response to mTBI.

### Therapeutic benefits of CtBP inhibition after mTBI

**Figure 4:**
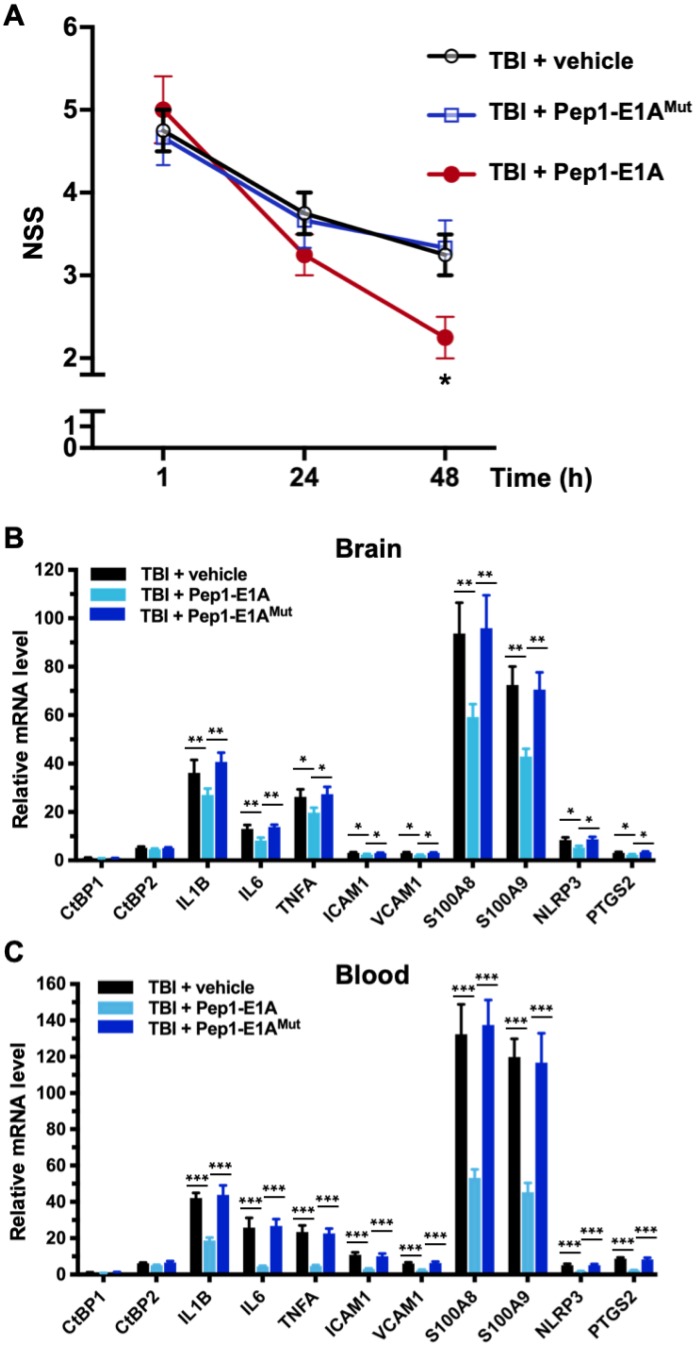
** Pep1-E1A reduces CtBP-dependent proinflammatory gene expression and ameliorates neurological deficits after mild brain injury.** Mice (*n* = 4 per group) received sham, a single 0.8 J TBI, or a single 0.8 J TBI followed by treatment with Pep1-E1A or Pep1-E1A^Mut^ (2 mg/kg, i.p.) at 1 h and 24 h after injury. **(A)** Comparison of NSS scores at 1, 24 and 48 h postinjury. Data are presented as mean ± SD from four animals and were analyzed among the three TBI groups by one-way ANOVA followed by Bonferroni's *post hoc* tests. Asterisk indicates significant difference between TBI + vehicle and TBI + Pep1-E1A mice at 48 h postinjury (*p* < 0.05; F_(2,9)_ = 5.33). **(B and C)** Comparison of mRNA expression of the CtBP target genes in brain **(B)** and peripheral blood leukocytes **(C)** at 48 h postinjury. Results (mean ± SD) were normalized to sham and analyzed by t-test. *n* = 4; *p<0.05, **p<0.01, ***p<0.001.

Because Pep1-E1A suppresses LPS-induced inflammatory response in primary microglia and astrocytes by directly targeting the transcriptional activation function of CtBP1 and CtBP2 (see Figures 2A and S1A), we investigated whether Pep1-E1A could be useful in attenuating the acute inflammatory sequelae of brain trauma using our mouse model of mild head injury. To assess the therapeutic efficacy of Pep1-E1A on head trauma, animals were subjected to the application of a single head impact of 0.8 J energy. The animals were then randomized to begin treatment with placebo (vehicle), Pep1-E1A or Pep1-E1A^Mut^ (3 mg/kg by i.p. injection at 1 h and 24 h postinjury). An evaluation of neurological function was performed at 1 h, 24 h and 48 h after injury using criteria of the neurological severity score (NSS). We found that averaged NSS numbers were statistically different between the vehicle control and Pep1-E1A-treated groups at 48 h postinjury (*p* < 0.05; F_(2,9)_ = 5.33), while no significant differences were observed between the vehicle control and Pep1-E1A^Mut^-treated groups (Figure 4A). As expected, Pep-E1A treatment significantly reduces mRNA expression of the nine CtBP-regulated proinflammatory genes described above near 48 h postinjury in both brain tissue and peripheral blood leukocytes (Figures 4A and 4B). Thus, these results demonstrate that the inhibition of CtBP1 and CtBP2 ameliorates mTBI-induced inflammatory damage in mice. It is also noteworthy that the inhibitory effect of Pep1-E1A on the CtBP-mediated transactivation is more pronounced in the peripheral blood cells (56-83%) than in the brain tissues (21-41%). One possibility for the differential CNS and systemic effects is the assumed incapability of the peptidic Pep1-E1A inhibitor to efficiently cross the blood-brain barrier.

**Figure 5:**
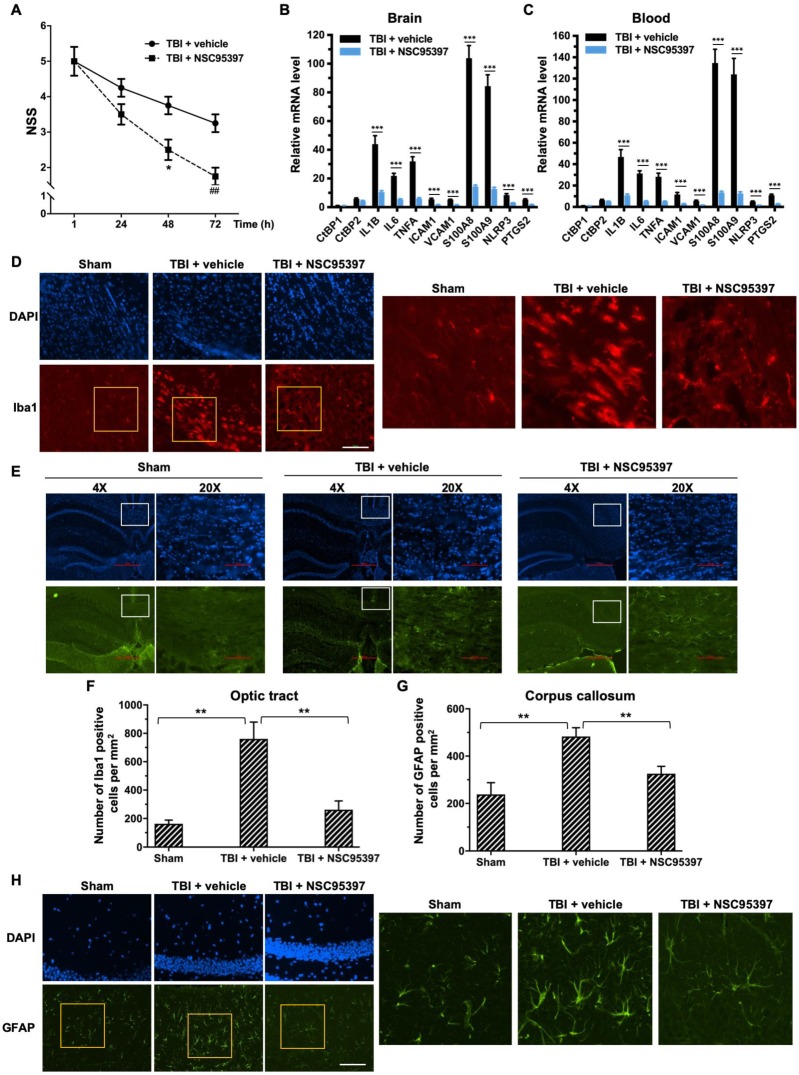
** Post-injury treatment with NSC93597 reduces microglia and astrocyte activation, suppresses transactivation of CtBP target genes and improves neurological outcome.** Mice (*n* = 4 per group) received a single 0.8 J TBI followed by i.p. injections of vehicle or NSC95397 (0.5 mg/kg) at 1 h, 24 h and 48 h postinjury. **(A)** NSS scores (mean ± SD) analyzed by t-test revealed significant effect of NSC95397 treatment at 48 h (*n* = 4; *p<0.05) and 72 h (*n* = 4; ## *p*<0.01).**(B and C)** Relative mRNA expression levels (mean ± SD) in brain **(B)** and peripheral blood leukocytes **(C)** at 72 h postinjury were normalized to sham and analyzed by t-test. *n* = 4; ***p<0.001. **(D)** Representative images of microglia visualized by Iba1 immunostaining (red) in the optic tract at 72 h postinjury. Nuclei were stained by DAPI. Scale bar, 100 μm. The three panels on the right are higher magnification images of the respective framed regions within the Iba1-stained panels on the left. **(E)** Representative images of astrocytes in the corpus callosum visualized by immunostaining for GFAP (green) as described in **(D)**.** (F and G)** Quantitation of the microglia and astrocyte response by counting the number of Iba1-positive **(F)** and GFAP-positive **(G)** cells per mm^2^ in the optic tract and corpus callosum regions, respectively. Data are presented as mean ± SD and analyzed by t-test. *n* = 3; **p<0.01.**(H)** Representative images of GFAP-positive astrocytes in the hippocampal CA1 region 72 h postinjury. Scale bar, 100 μm. Higher magnification images of the respective framed regions within the GFAP-stained panels on the left are shown in panels on the right.

The small molecule NSC95397, like the peptide Pep1-E1A, inhibits the transcriptional repressor activity of CtBP1 and CtBP2 by interfering with their binding to PXDLS-containing targets (Blevins et al., 2015). The above mouse model of mild head injury was used to examine the effectiveness of NSC95397 in limiting neuronal damage following TBI. NSC95397-treated mice showed a significant improvement in neurobehavioral deficits, and lower NSS compared to the vehicle control at 48 h (*p* < 0.05) and 72 h (*p* < 0.01) postinjury (Figure 5A). NSC95397 demonstrated equal inhibitory effects on the expression of the nine CtBP target genes in brain tissue (66-86%) and peripheral blood leukocytes (68-90%) at 72 h postinjury (Figures 5B and 5C).

We next examined the effects of NSC95397 on the response of microglia and astrocytes to local brain injury at 3 d after mTBI by immunofluorescence staining using antibodies specific for the microglial marker Iba1 and the astrocytic marker GFAP 62, 63. Compared to sham brains, injured brains showed significant increases in the number of Iba1-positive microglia in the optic tract and of GFAP-positive astrocytes in the corpus callosum, indicating the activation and proliferation of these CNS glial cells following head injury (Figure 5 D-G). By contrast, we observed significantly reduced numbers of Iba1-positive microglia and GFAP-positive astrocytes in the two above white matter-rich regions of NSC95397-treated brains (Figure 5 D-G). On the other hand, Iba1-positive microglia in the optic tract of the injured brain exhibited the amoeboid-like morphology that is typically associated with activated microglia, while Iba1-positive microglia in the NSC95397-treated brain showed decreased cell soma volume and increased cell ramification, largely resembling the resting state morphology in the sham brain (Figure 5D). In addition to white matter-rich regions, we also noted that NSC95397 treatment features GFAP-positive astrocytes in the hippocampal CA1 region with smaller, more compacted cell bodies and elaborated thinner processes as compared to the vehicle control group (Figure 5H). These results support our hypothesis that the pharmacological inhibition of CtBP1 and CtBP2 is a promising strategy for the treatment of brain injury-induced inflammation and neurodegeneration.

**Figure 6:**
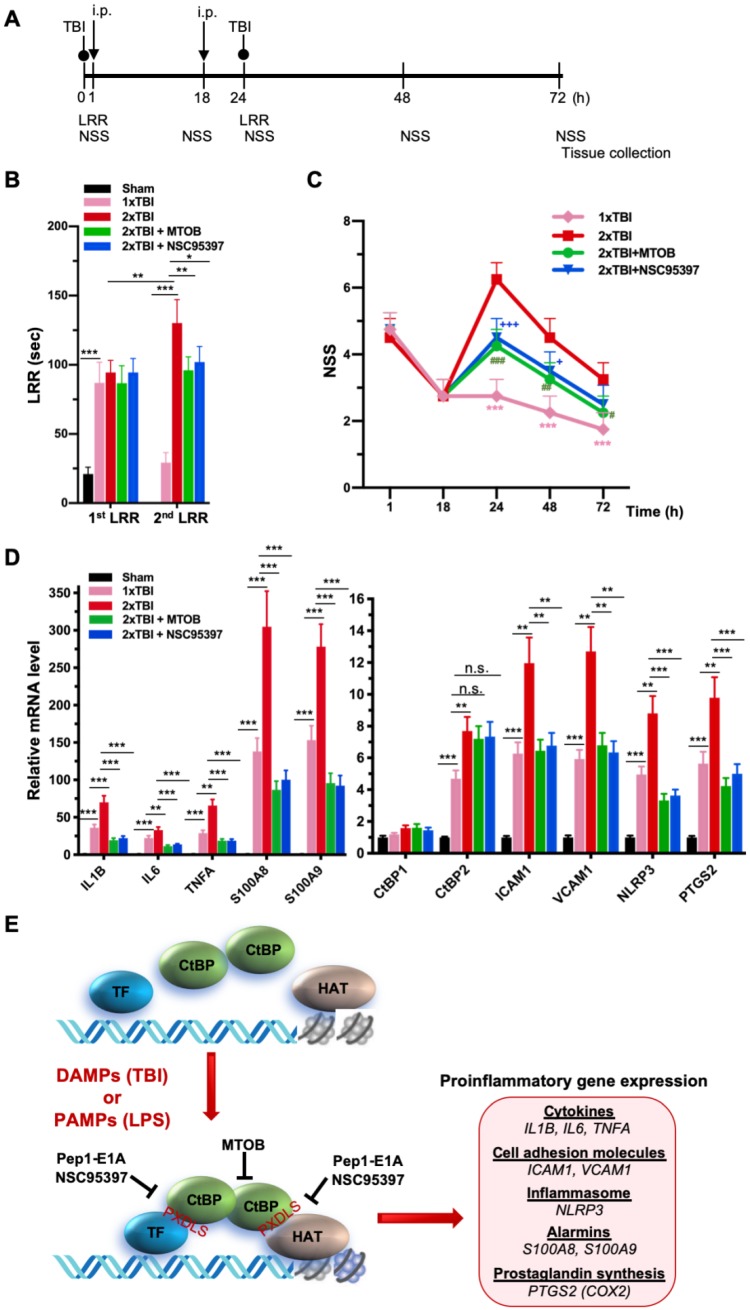
** NSC95397 and MTOB attenuate neuroinflammation caused by repetitive mild TBI. (A)** Experimental timeline. Mice (*n* = 4 per group) received a single head impact of 0.5 J energy (1xTBI), or two 0.5 J impacts (2xTBI) spaced 24 h apart. The CtBP inhibitor-treated groups were given an i.p. injection of MTOB (860 mg/kg) or NSC95397 (1.5 mg/kg) at 1 h and 18 h after the first injury. **(B)** LRR durations (mean ± SD) following the first and second head injury were analyzed by t-test. *n* = 4; *p<0.05, **p<0.01, ***p<0.001. **(C)** CtBP inhibitors improved neurological deficits in mice receiving repeated mTBI. NSS assessment at 1 h and 18 h was given prior to the administration of the CtBP inhibitors. NSS scores (mean ± SD) were analyzed by two-way ANOVA with Bonferroni *post hoc* test of significance between individual groups. Asterisk indicate a significant difference between 1xTBI and 2xTBI (***p<0.001) mice; hash symbol indicates a significant difference between 2xTBI and 2xTBI with MTOB treatment, (^#^*p*<0.01, ^##^*p*<0.01, ^###^*p*<0.001); plus symbol indicates a significant difference between the 2xTBI and 2xTBI + NSC95397 groups (^+^*p*<0.01, ^+++^*p*<0.001). **(D)** NSC95397 and MTOB prevent a further increase in the mRNA expression levels of CtBP target genes in the animal brains experiencing repeated TBI. Brain tissues were collected for mRNA analysis at 72 h after the first injury; results (mean ± SD) were normalized to sham and analyzed by t-test. *n* = 4; **p<0.01, ***p<0.001. **(E)** A model for CtBP-mediated transactivation in inflammatory response and immune activation.

As noted above, another small-molecule CtBP dehydrogenase inhibitor, MTOB, has been shown to antagonize the transcriptional regulatory activity of CtBP1 and CtBP2 by eviction from their target promoters in breast cancer cell lines 58. We therefore investigated whether MTOB, as well as NSC95397, could alleviate neuroinflammation caused by repetitive mild head injury. Mice were given either a single injury (1xTBI) or two injuries 24 h apart (2xTBI). The 2xTBI mice received an injection of placebo (vehicle), MTOB (860 mg/kg) or NSC95397 (1.5 mg/kg) at 1 h and 18 h after the first injury (see Figure 6A for experimental timeline). All mTBI groups showed significantly increased loss of righting reflex (LRR) duration following the first injury as compared to sham mice (Figure 6B). The 1xTBI group regained righting reflex within 24 h after injury, and the 2xTBI group had a significantly longer LRR duration following the second injury (*p <* 0.01) (Figure 6B). The increased duration of righting reflex was effectively suppressed by administration of MTOB (*p* < 0.01) or NSC95397 (*p* < 0.05) after the first injury (Figure 6B). Furthermore, the 2xTBI group exhibited significantly higher NSS than the 1xTBI group at 24 h, 48 h and 72 h (*p* < 0.001) following the first injury (Figure 6C). NSS scores were significantly decreased by MTOB (*p* < 0.001 at 24 h; *p* < 0.01 at 48 h; and *p* < 0.05 at 72 h) and by NSC95397 (*p* < 0.001 at 24 h and *p* < 0.05 at 48 h) (Figure 6C). In addition, we observed a 150 to 230% increase in the mRNA expression of the nine CtBP-regulated proinflammatory genes in the brain tissues of the 2xTBI group as compared to the 1xTBI group at 3 d after the first injury (Figure 6D). The inhibitor-treated mice exhibited lower or equal levels of expression of these CtBP target genes relative to the 1xTBI mice (Figure 6D). Therefore, MTOB and NSC95397 attenuate repetitive head injury-elicited neurologic dysfunction via inhibition of the transactivation activity of CtBP1 and CtBP2.

In summary, we prove that CtBP1 and CtBP2 can transactivate select proinflammatory target genes in single and repetitive mTBI mice, thus contributing to acute neurological deficits resembling clinical mTBI symptoms that can be quantified using the NSS score. We have also demonstrated that postinjury treatment using three distinct CtBP inhibitors with different mechanisms of action have similar effects on suppressing CtBP-controlled proinflammatory gene expression (see the model in Figure 6E), mitigating the TBI-induced inflammatory pathology and significantly improving mouse brain function in the acute-to-subacute phase. Further studies are needed to characterize cell type specific function of CtBPs and the long-term consequence of modulating CtBPs' activity in the mTBI mice. Finally, and most importantly, these findings suggest that suppression of CtBP function could potentially be an effective strategy to alleviate TBI-induced inflammation and neurological impairment.

## Supplementary Material

Supplementary figures and table.
