# Supplementary material for: C-terminal binding proteins 1 and 2 in traumatic brain injury-induced inflammation and their inhibition as an approach for anti-inflammatory treatment
Source: Int J Biol Sci. 2020 Feb 4;16(7):1107–20. doi: 10.7150/ijbs.42109 (PMC7053329; doi:10.7150/ijbs.42109)
Supplement: Supplementary file 1 — Supplementary figures and table. [file ijbsv16p1107s1.pdf]

SUPPLEMENTARY MATERIALS

C-terminal binding proteins 1 and 2 in traumatic brain injury-induced inflammation and their inhibition as an approach for anti-inflammatory treatment

Hong Li[1]*, Caiguo Zhang[1]*, Chunxia Yang[1,2], Melanie Blevins[3], David Norris[1], Rui Zhao[3], and Mingxia Huang[1,#]

Figure S1

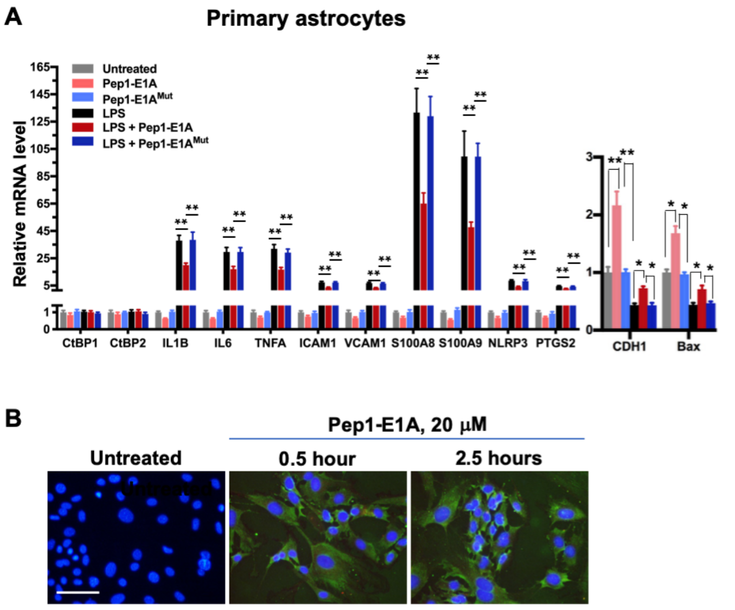

Figure S1. Pep1-E1A suppresses LPS-induced inflammatory gene expression in mouse primary astrocytes.

(**A**) Effects of pretreatment with 20 µM Pep1-E1A or Pep1-E1A^{Mut} on the basal and LPS-induced mRNA expression of CtBP target genes in mouse primary astrocytes. n = 3; *p<0.05, **p<0.01, ***p<0.001.

(**B)** Representative immunostaining images of Pep1-E1A internalization into cultured astrocytes after 0.5 and 2.5 h incubation. Scale bar, 50 µm.

Figure S2

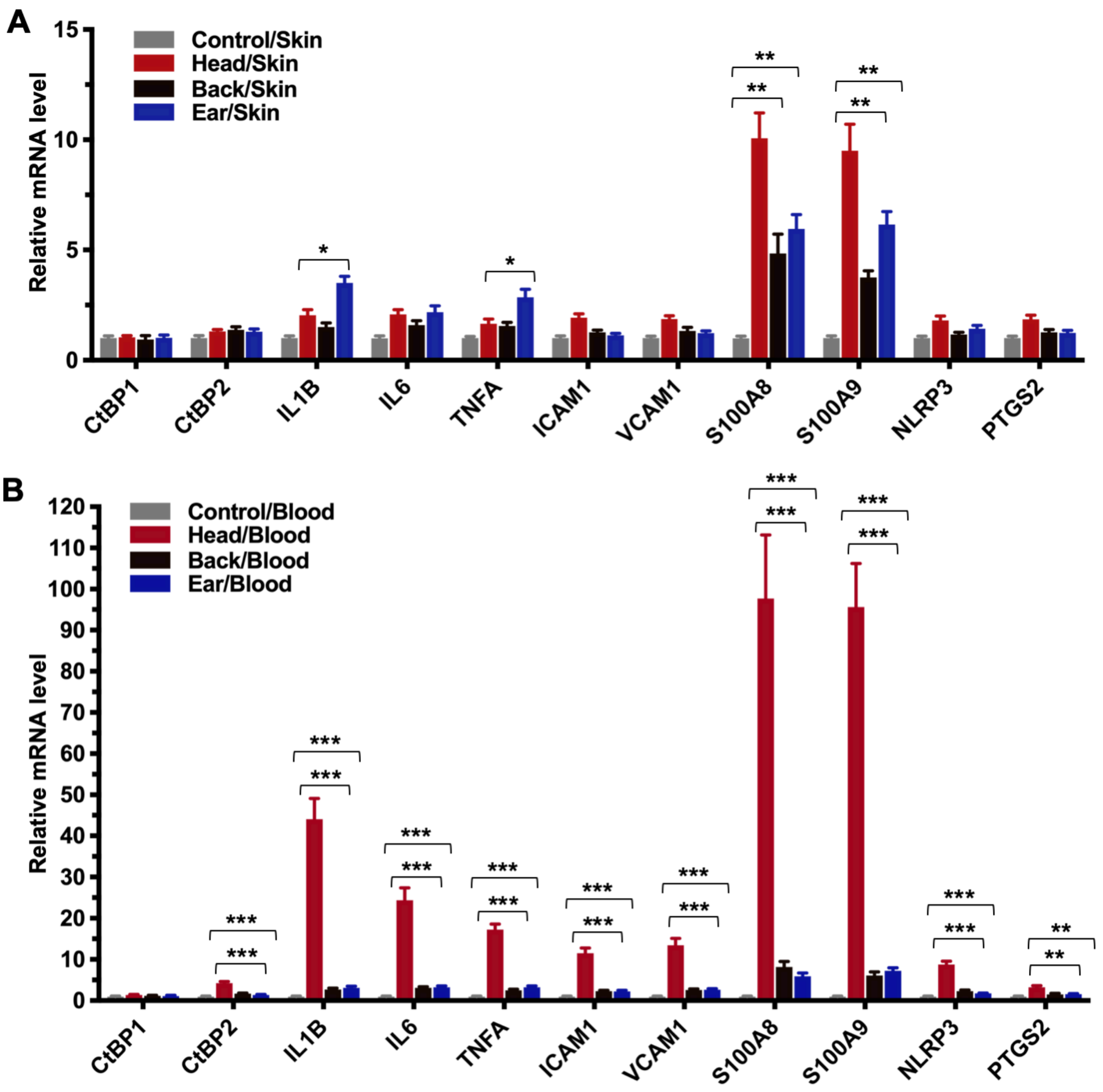

Figure S2. **CHIMERA-delivered mild TBI causes systemic inflammatory response.**

Mice received sham procedure (control) or a single impact of 0.7 J energy to the head, the back, or the ear. Skin tissues containing the site of the impact (**A**) and peripheral blood leukocytes (**B**) and were collected 24 h postinjury for total RNA extraction and RT-qPCR analysis. $n = 4$; *$p<0.05$, **$p<0.01$, ***$p<0.001$.

Table S1. Sequences of primers used in this study.

**ChIP-qPCR**	**Forward** (5' to 3')	**Reverse** (5' to 3')
mIL1B	ACATGAGACTGGCTATGGTATT	TTATCCCTTTTCCAGGTCTCCC
mIL6	AGGGCTAGCCTCAAGGATGACT	GGAGTCAACTCTCTAATTTTGA
mTNFA	CAGCCCTCCCAAAGCCCATGCA	GTGCTTCTGAAAGCTGGGTGCA
mS100A8	GCCTAGACATGGACTTATTGCC	AGCATTTCTGAGTCTGAGGAAG
**RT-qPCR**		
mCtBP1	TAGGCGGGGCAAGAGGAAGC	CGAGGAACGCAAAGGACACAGG
mCtBP2	GGCACACCCCTCCCAAGCTC	GGCACACCCCTCCCAAGCTC
mIL1B	GCCCATCAGAGGCAAGGAGGA	CAGGTCGCTCAGGGTCACAAGA
mIL6	TGGCTAAGGACCAAGACCATCCA	CATAACGCACTAGGTTTGCCGAGTAGA
mTNFA	CCCACACCGTCAGCCGATTT	CCTTGGGGCAGGGGCTCTT
mICAM1	TGGTACATACGTGTGCCATGCCT	ATCCTGATCTTTCTCTGGCGGT
mVCAM1	TCCGCCAGGCACAGCTGCAGGA	ATTACCAAGGAAGATGCGCAG
mS100A8	TCGAGGAGTTCCTTGCGATGG	ACATATCCAGGGACCCAGCCCTA
mS100A9	TGGAGGACCTGGACACAAACCAG	TTCCCACAGCCTTTGCCATGA
mNLRP3	CCCCCTCCTCCTCCGCTTCT	CTAGCCCACGGGCAGCTCCT
mPTGS2	GGACTGCAGAAGGCCCCATGT	GGAACACAGCTACGAAAACCAATCA
mCDH1	CCCCTCCAATGCCTGCTCTTG	TCTGACTGCCTCTGCCTCCTGA
mBax	AGTCCTCACCGCCTCGCTCA	GCCTTTCCCCTTCCCCCATT
